# Characterization and drug susceptibility pattern of *Salmonella* and *Shigella* in children below five years: a cross-sectional study conducted in Lodwar, Turkana County, in Northern Kenya

**DOI:** 10.11604/pamj.2022.42.13.32025

**Published:** 2022-05-09

**Authors:** Simion Kipchirchir Leting, Stanslaus Kiilu Musyoki, Geoffrey Kattam Maiyoh

**Affiliations:** 1 Department of Medical Laboratory Sciences, School of Health Sciences, Kisii University, Kisii, Kenya,; 2 Department of Laboratory, Diagnostic Unit, Lodwar County and Referral Hospital, Lodwar, Kenya,; 3 Department of Biochemistry and Clinical Chemistry, School of Medicine, Moi University, Eldoret, Kenya

**Keywords:** Drug susceptibility, *Salmonella and Shigella*, children

## Abstract

**Introduction:**

Salmonella and Shigella infections are waterborne associated infections globally known to cause serious illnesses in all age groups, but can be more devastating in children below five years. Antimicrobial resistance has been known to worsen the existing challenge in the management of Salmonella and Shigella infections. The aim is to isolate and identify Salmonella and Shigella among children less than five years with diarrhea and to determine resistance to commonly prescribed drugs at the Lodwar County and Referral Hospital in Northern Kenya.

**Methods:**

using a cross-sectional study design, a descriptive experimental study was conducted on 196 children with diarrhea using rectal swabs. A structured questionnaire was used to collect sociodemographic information. Samples were then received in the microbiology laboratory, and macroscopic and microscopic examinations were done before culture on specific selective media. Thereafter, biochemical confirmation of the growths done then confirmed results tabulated before analysis.

**Results:**

from the total samples collected (196) Shigella dysenteriae cases were 4 (5%), while Shigella Flexneri were 7 (9%), Shigella sonnei were 3 (4%), Shigella boydii were 4 (5%) and Salmonella typhimurium were 2 (2.4%). From these, about 70% of the isolated Salmonella and Shigella demonstrated high antibiotic resistance to Amoxilliclav and Ampicillin, both with high minimum inhibitory concentrations (MICs) values of about 8ug/ml. While over 80% drug susceptibility was noted in Amikacin (1ug/ml), Ciprofloxacin (2ug/ml), Ceftriaxone (4ug/ml) and Ceftazidime (4ug/ml).

**Conclusion:**

Salmonella and Shigella are among the common contributors of diarrhea among children less than five years. Drug resistance among the commonly used antibiotics is a serious indicator that possible misuse of antibiotics especially the beta lactam penicillin's.

## Introduction

*Salmonella* and *Shigella* are a global public health threat and a cause of foodborne and or waterborne related diseases. According to World Health Organization (WHO), 2 billion people still do not have basic sanitation facilities like toilets and latrines and out of these 673 million still defecate in the open places like bushes and near water bodies [1]. In Kenya, among the 47 counties, Turkana was ranked as the leading in open defecation rates [2]. These poor sanitation facilities are directly linked to the transmission of infections e.g. *salmonella* and *Shigella* among others which eventually cause diarrhea and diarrheal related conditions. Globally, *Salmonella* and *Shigella* are common diarrheagenic pathogens, among others not studied here. These infections are more predominant in children under five years of age. They are associated with high mortality and morbidity in developing countries. Salmonella belongs to two major groups depending on the disease it causes; one is the enteric fever group which causes typhoid fever, and it consists of typhoid and paratyphoid bacilli. The second group is the non-typhoid *Salmonella* which include *Salmonella typhimurium* and *Salmonella enteritidis* among many other serovars causing gastrointestinal infections. *Shigella species* are subdivided into four serogroups, groups A, B, C and group D. *Shigella dysenteriae, Shigella flexneri, Shigella boydii* and *Shigella sonnei* belong to groups A, B, C and group D respectively. *Shigella sonnei*is the most prevalent species in developed countries, while *Shigella flexneri* tends to be less predominant. Two rare species as compared to the rest include *Shigella dysenteria* and *Shigella boydii* since they cause shigellosis which is endemic in most developing countries and a major cause of bloody diarrhea worldwide [3].

*Salmonella serovars* habitate in faecally contaminated food, livestock and poultry products notably eggs and egg products [4] while *Shigella* is also known to naturally habitate in man, hence man acting as a reservoir, is commonly transmitted via contaminated food or water a mechanism commonly known as fecal-oral route. The conditions caused by these bacterial pathogens are endemic in developing countries where hygiene and sanitation is poor [5]. Due to scarcity of clean treated water [6], the existence of non-hygienic practices including open defecation [7] coupled with the increase in diarrheal cases of children attending Lodwar County and Referral Hospital (LCRH), triggered further research and investigations. Hence, due to the documented high levels of open defecation and water scarcity in the region coupled with high cases of diarrheal cases among patients attending LCRH, the present study was designed to investigate the roles of Salmonella and Shigella infections among diarrheal children attending this regional health facility. Based on literature search, no similar study has been conducted in Lodwar to date. The rise in the development of drug resistance among bacterial infections is also becoming a global concern despite the numerous studies across the world, this is attributed to misuse and overuse of antibiotics both by human, animal and environmental health [8] this is very rampant among developing countries where resistance to routinely used antimicrobial agents has been witnessed. The rise in drug resistance has also been associated with poverty, since people's options are limited because of the limited resources. High unhygienic levels, therefore, are likely because of the inability to buy sanitation products including access to clean water hence increased chances of infection, also the inability to access healthcare or buy drugs [9]. The objective of this study is to determine the prevalence and drug susceptibility of *Salmonella* and *Shigella* among diarrheal children under five attending Lodwar Referral Hospital in Turkana, Northern Western Kenya.

## Methods

**Study subjects:** this research study which involved 196 study samples was carried out at Lodwar County and Referral Hospital in Turkana County, Kenya. Sample collection was conducted between 1^st^ January to 30^th^ September 2019. The study mainly focused on new diarrheal cases, where a majority of them were randomly recruited at the outpatient and some through the pediatric ward. The study population involved children below five years who attended LCRH hospital and met the pre-set inclusion criteria; i.e. assent, frequency of two to three loose bowel movements within the last 24 hours, should not be on antibiotics at the time of sample collection, and those below five years of age.

**Stool sample collection:** sample collection was accomplished in two ways; by use of a stool polypot or a rectal swab was used as an alternative. This is because it was realized that most children were unable to get a stool sample at that time. The parent or guardian of a child was provided with a sterile stool polypot and a sterile swab for either stool or rectal swab respectively; oral instructions on sample collection were then given to each client, during this time demographic information was captured by filling a questionnaire upon delivery of the sample it was then received and taken to the microbiology laboratory for analysis. After inoculation, all samples were preserved for a period of 24 hours for retrieval in case of need.

### Isolation and identification

**Stool culture:** at the microbiology lab, stool samples were inoculated in xylose lysine deoxycholate (XLD) and deoxycholate citrate agar (DCA) media already prepared from agar using manufacturers´ standard instructions. The inoculated culture plates were then incubated at between 35°C- 37°C for up to 24 hours. Additionally, inoculation was done on selenite F for enrichment purposes. Upon growth, characteristic colonies were picked; small red colonies on XLD, others may have a blackening appearance; on DCA, colorless colonies some with black centers were picked for gram staining [10] as biochemical tests including Triple sugar Iron (TSI), urea, citrate, oxidase were done for confirmation purposes. American type culture collection (ATCC) bacterial strains were used as controls in processing the samples.

### Identification of *Salmonella* and *Shigella spp* by phenotyping

**Stereotyping of *Salmonella spp*:** first, to detect the presence of a *Salmonella* antigen, commercially available O and H antisera were used [11]. A clean glass slide was prepared by bordering and partitioning into three parts with a glass pencil. A drop of polyvalent was then put on the glass slide, a live bacterial colony was then picked and dispersed on the slide to form a homogenous suspension. The glass slide was tilted back and forth then the agglutination pattern was observed with a naked eye, a very strong agglutination was regarded as positive. Where there was no agglutination with the polyvalent sera, the same procedure was repeated using V1 antisera, where a positive reaction was found with V1 antisera a dense suspension of the organism was prepared using saline then heated to 121°C for 15 min then repeated using polyvalent sera and the V1 antiserum. Live bacterial cells from overnight cultures that agglutinated strongly with polyvalent sera within 1 min were regarded as positive; hence the heat-treated isolate that produced a distinct positive reaction with polyvalent sera was therefore interpreted as *Salmonella*. Where live cells produced a negative result with polyvalent sera and positive with V1 antiserum while the heated cells produce the contrary, such a specimen was regarded as *Salmonella typhi* [12]. To further confirm the *Salmonella* serotype, the Kauffman white classification scheme was applied [13].

***Shigella* serotyping:** serological identification was performed typically by slide agglutination with polyvalent somatic (O) antigen grouping sera A to D. Where necessary i.e. when agglutination is noted with the polyvalent, testing with monovalent antisera for specific serotype identification was also done to confirm the specific serotype. Typical *Shigella* isolates that agglutinated poorly or fail to agglutinate at all were retested using heated bacterial suspension. A heavy suspension of the isolates was prepared in a tube containing 2ml of normal saline solution; it was then placed in boiling water for 30 minutes. The suspensions were tested to ensure that they were not rough, then retested for agglutination in antisera A to D [14]. *Escherichia Coli* ATCC 25922 bacterial strains were used as a reference strain throughout the analysis, i.e. culture and antimicrobial susceptibility testing.

**Determination of antimicrobial susceptibility patterns:** once the specific bacterial pathogenic bacteria have been isolated and identified, the appropriate antimicrobial was selected according to the Clinical and Laboratory Standards Institute guideline (CLSI). Antimicrobial susceptibility was done through the Kirby bauer disc diffusion technique. Drug susceptibility procedure begun with the preparation of solid culture media known as Muller Hinton where it was prepared according to the manufacturer´s instructions. The media prepared was then poured while still in liquid form into sterile Petri dishes 15 to 20ml each, then allowed to solidify at room temperature. Once the Muller Hinton Agar was ready, the culture plates were preserved in a refrigerator if they are not used immediately. A pure culture of the isolated organism was then swabbed with a bacterial suspension of 0.5 McFarland standards using a sterile swab by smearing uniformly on the solid Muller Hinton Agar. Antimicrobial single discs with specific concentrations measured in microgram (ug) were then introduced into the media. Upto 10 different antimicrobial discs were placed on a single plate of Muller Hinton plate at a time. After that, the culture plates are incubated at 37°C for upto 24 hours.

**Determination of minimum inhibitory concentrations (MIC) and minimum bactericidal concentrations (MBC):** a two fold serial dilution was pipetted in a 96 well micro dilution plate, where a loopful of a bacterial colony from pure isolates was picked and a 0.5 McFarland standard was prepared from it. A bacterium inoculum was then dispensed into the micro dilution plate. A double dilution formula was applied in the preparation of the reducing concentration of the antibiotic being used from the original concentration to a reducing concentration up to 10 fold. The inoculated wells were covered with a lid or plate sealing tape and incubated at 37°C for upto 24 hours. Two control wells were included, the inoculated and the uninoculated, the first quality controls the adequacy of the broth onto support bacterial growth while the second checks for the sterility of the well. Finally, the micro dilutions were read to determine MIC concentration, and inoculation on nutrient agar was done to determine MBC points. The antimicrobial drug discs used in this study included the commonly used antibiotics in the treatment of the diseases. These included; amoxilliclav (AUG 30ug), gentamicin (CN), amikacin (AK 30ug) ampicillin (AMP 10ug), trimethoprim (SXT 25ug), ceftriaxone (CRO 30ug), ceftazidime (CAZ 30ug), ciprofloxacin (CIP 5ug) and trimethoprim (SXT 25ug). After incubation, the zone of clearance was measured in millimeters (mm) and documented. Zones of inhibition was then measured (in mm) and the results were recorded as either sensitive (s), intermediate (I) or resistant (R) based on WHO Drug information and Clinical and Laboratory Standards Institute [15]. After completion of sample analysis, all data were tabulated in a Microsoft Excel sheet pending further analysis.

**Data quality:** a unique number was given upon labeling to avoid mix-up in case of name duplication, the same details were then transferred to a register where results were recorded, where proper labeling of the sample was not done, and the sample was rejected immediately. Quality assurance procedures were adhered to at each step of the process i.e. during media preparation, calibrated equipment was used (autoclave, weighing scale, incubator), checking of expires in all reagents, storage conditions and finally the use of certified organisms to check viability, sterility testing of media. Finally, all the laboratory analysis done was run against known control bacteria (ATCC strains). Data was thereafter transferred to an Excel sheet in a computer, ensuring that all information is updated and consistent with the findings in.

**Sample size:** a sample size of 196 study cases was used in this research study. Using p as the estimated proportion of the aspect of interest in that population at 0.5 indicating maximum variability, the sample size found was noted to be larger (385) hence more expensive. To further reduce the sample size with 5% margin of error, and using finite calculation formula, a sample size of 196 was arrived at.

**Ethical considerations:** immediately before sample collection, the client had to give assent from his parents; ethical approval was sought from the Institutional research and ethics committee (IREC) of Moi University while permissions were obtained from the National Commission for Science Technology and Innovation and the County government of Turkana, Kenya. To safeguard client information, all samples were given a unique identifier or code.

## Results

**Demographic characteristics of the study participants:** out of the total suspects of 196 who met the predefined inclusion criteria, stool samples were collected and analyzed; (95) 48.5 percent were females while (101) 51.5 percent were males. In terms of age category, (152) 77.6 percent of the children were under 2 years of age while (44) 22.4 percent are above 2 years (Table 1). The level of education of the mothers/guardian was also assessed, and it was noted that most of the mothers coming with their children with diarrheal complaints were as follows; those whose level of education were college level and above were 5.6%, secondary education were 20.4%, primary education were 26% while those found to be who are totally illiterate were at 48%. Their source of water was also captured and 61.7% of participant parents obtain their water from a borehole or lager while only 38.3% were connected to piped water. All this demographic information is as indicated in Table 1. Out of the 196 stool samples collected from the study participants, specifically children under five years of age; *Shigella spp* was the most common pathogenic organism isolated, followed by *Salmonella spp* with an average prevalence of 9.2% and 1% respectively. The positivity rate was as follows; 35% *Shigella flexneri*, 20% *Shigella dysenteriae*, 20% *Shigella boydii* 15% *Shigella sonnei* and while *Salmonella spp* was least detected at 10 percent (*Salmonella typhimurium*). The breakdown of this data is as tabulated below (Table 2).

**Table 1:** demographic information of the study subjects

Characteristic/item		Total number screened	Percentage (%)
Ages	infants < 2 yrs ( 24 months)	133	66.8
	Children ≥2yrs	66	33.2
Gender	Males	103	51.7
	Females	9	48.3
Water sources	Piped	76	38.2
	Borehole/lager	123	61.8
Mothers level education	Never went to school	95	47.7
	Primary	52	26.1
	Secondary	41	20.6
	Tertiary	11	5.5
Duration of diarrhoea	<3episodes	48	24.1
	≥3 episodes	151	75.9
Stool consistency	Watery	26	13.1
	Mucoid	169	84.9
	Blood stained	4	2

**Table 2:** distribution of enteric bacteria isolated

Micro organism	Number of cases (n)	Percentage (%)
*Shigella dysenteriae*	4	20
*Shigella flexneri*	7	35
*Shigella sonnei*	3	15
*Shigella boydii*	4	20
*Salmonella typhimurium*	2	10

**Figure 1:**
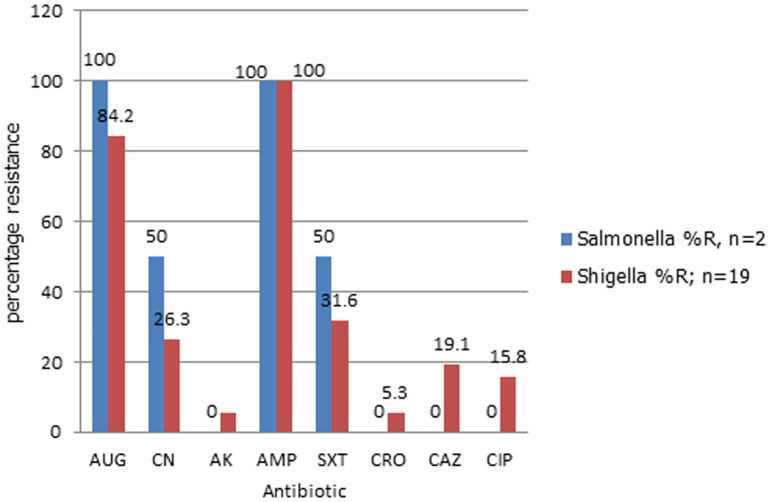
graph showing percentage resistance of *Shigella* and *Salmonella spp* against various antimicrobials

**Drug resistance report:** drug susceptibility was carried out to determine the antimicrobial resistance trends for all the isolated pathogenic microorganisms. After incubation, the zone of clearance was measured in millimeters (mm) and documented. The results as shown in Figure 1 were as follows; Ampicillin was (21)100% resistant to both *Salmonella* (2) and *Shigella* (18) isolated. Second most resistant drug with *Shigella spp* was Amoxilliclav at (16)84.2%, followed by trimethoprim (6)31.6%, gentamicin at (5)26.3%, ceftazidime at (17)19.1% then ceftriaxone and amikacin both at 1(5.3%). For *Salmonella spp* apart from Ampicillin at 100% resistance, Amoxilliclav are also (2)100% resistant. Gentamicin and trimethoprim are 50% resistant to *Salmonella spp* while amikacin, ceftazidime and ceftriaxone are all (0) 0% or non-resistant to *salmonella spp*.

**Minimum inhibitory concentration (MIC) and minimum bactericidal concentration (MBC):** from the study, the average MIC and MBC values for *Salmonella* and *Shigella* species is as shown in the Table 3.

**Table 3:** data on minimum inhibitory concentrations (MIC) and minimum bactericidal concentrations (MBC) determination of salmonella and shigella isolated

Antibiotic	*Shigella spp*		*Salmonella spp*	
	Average MIC	Average MBC	Average MIC	Average MBC
Augmentin	≤8	≤4	≤8	≤4
Gentamicin	≤4	≤2	≤4	≤2
Amikacin	≤1	≤1	≤1	≤0.5
Ampicillin	≤8	≤4	≤8	≤4
Trimethoprim	≤4	≤2	≤4	≤2
Ceftriaxone	≤2	≤1	≤2	≤1
Ceftazidime	≤4	≤2	≤4	≤2
Ciprofloxacin	≤2	≤1	≤2	≤1
Positive control	2	2	2	2
Negative control	0	0	0	0

## Discussion

**Statement of principal findings:** from this study of a sample size of 196, the demographic information demonstrate that majority of children affected and infected are below 2 years of age, this could be as a result that most of them are still at the weaning stage. The study findings correspond to a similar cross-sectional study on diarrheal children under five conducted in Southern Ethiopia [16] where the prevalence of *Shigella spp* was 7.6% while *Salmonella spp* at about 1%. *Salmonella spp* was the least isolated among diarrheal children at 2%. The high prevalence of *Shigella spp* as compared to *Salmonella spp* could be *Shigella* has a very low infectious dose hence transmission rate is high. Among the *Shigella spp* the most prevalent of them is *Shigella flexneri* followed by *Shigella dysenteriae* while the least is *Shigella sonnei*. The breakdown in *Shigella spp* prevalence closely corresponds to a study conducted in Ethiopia [17] where *Shigella flexneri* (45%) was most prevalent followed by *Shigella dysenteriae* (30%) then *Shigella boydii* (25%). This correspondence could be attributed to regional and climatic similarities in the region since Ethiopia borders Northern Kenya. It is common among the malnourished children and in resource limited areas where sanitation and hygiene is poor.

**Discussion of important differences in results**: among the isolated *Shigella spp, Shigella flexneri* is less virulent as compared with the rest while *Shigella dysenteriae* is the most virulent. A study conducted in central Kenya at the Kiambu County Hospital partly corresponds on the *Shigella* isolation where *Shigella flexneri* was the most prevalent, while in contrast *Shigella sonnei* was the second most prevalent while *Shigella flexneri* was the least. Contrary to a number of studies conducted in developing countries where *Shigella flexneri* is the most prevalent, in third world countries, the opposite is true, this is confirmed by a study in Iran [18] and another study in the United Kingdom (UK) indicating the dominancy of *Shigella sonnei* since in industrialized nations they have a better system of access to healthcare therefore frequent exposure to antibiotics renders the other species of *Shigella* to clear, but *Shigella spp* has an unusual characteristic of acquisition and sustenance of antibiotic drug resistance genes from other bacterial species [19]. Another explanation to this *Shigella sonnei* and *Shigella flexneri* controversy is that *Sonnei* prevalence in developing countries is low because the entire population has a natural kind of immunity acquired through cross protection due to exposure to a certain group of micro-organisms called *Plesiomonas shigelloides* found in contaminated water supplies while in developed countries *P.shigelloides* is rarely found because the water supplies are clean and free of bacteria [19]. With this kind of scenarios, it is worth noting that the safety achieved through clean quality water, a reverse mechanism of antimicrobial drug resistance is likely to develop especially of *Shigella sonnei* and therefore it is important to introduce a vaccine possibly against this stubborn pathogen. *Shigella spp* were found to be 100% resistant to Ampicillin, it corresponds to a scientific study conducted in Lilongwe, Malawi [5] where *Shigella* isolates were fully resistant to both Ampicillin and Trimethoprim (at 100%). In speculation, the isolates could be carrying similar resistant genes since the conditions favouring them thrive are almost similar. *Salmonella spp* isolated was 100% resistant to Ampicillin and Amoxilliclav. This corresponds to a study in Burkina Faso [20] where Ampicillin and Amoxilliclav were highly resistant at 96.2% and 92.5% respectively. The high level of resistance in these studies could be a result of misuse and overuse through self-medication from over the counter.

**Limitations of the study:** the study was confined specifically to *Salmonella* and *Shigella* leaving out other pathogenic causes of diarrhea i.e. bacterial, viral, and fungal pathogens. With more funding, an extensive research can be conducted to ascertain the extent to which these infections impact on the lives of children below five. Another shortcoming is the lack of prior similar study in the same locality to support the background knowledge to lay a foundation to better understand the research problem. Additionally, since the study was conducted from one facility, hence impossible to generalize findings to the entire surrounding population. Finally, diagnostic analysis was only based on phenotypic and not molecular of which though expensive is more sensitive hence gives a detailed analysis and a wide range of possible etiologies.

## Conclusion

From this study, it is worth noting that *Salmonella spp* and *Shigella spp* are in part, among the causative agents of diarrhea among children attending LCRH with *Shigella* being more predominant in comparison to *Salmonella* infections. Data from this study demonstrate that both salmonella and *Shigella* isolated have developed a multidrug resistance to penicillin and penicillin related antibiotics, in this case Ampicillin and Amoxilliclav. These drugs therefore cannot qualify to be used in treatment of diarrheal infections in this facility. The best antibiotics of choice include; amikacin, ceftriaxone, ceftazidime and ciprofloxacin all with over 80% efficiency.

### What is known about this topic

- Poor hygienic conditions enhance transmission of bacterial infections, including Salmonella and Shigella;
- Children below five years have a poorly developed immune system hence risk of a wide range of infections.

### What this study adds

- Through this study, it is now known that ampicillin and amoxilliclav should not be prescribed on Salmonella and Shigella infections as both are unable to clear these infections; on the other hand, ceftriaxone, ciprofloxacin, ceftazidime and amikacin shall be the drug of choice in the treatment of these infections.

## References

[1] World Health Organization (2019). Sanitation.

[2] Njuguna J, Muruka C (2017). Open defecation in newly created kenyan counties: a situational analysis. J Health Care Poor Underserved.

[3] Shad AA, Shad WA (2021). Shigella Sonnei: virulence and antibiotic resistance. Arch Microbiol.

[4] Aung Kyaw, Khor Wei, Octavia Sophie, Ye Agnes, Leo Justina, Chan Pei Pei (2020). Distribution of *Salmonella* serovars in humans, foods, farm animals and environment, companion and wildlife animals in Singapore. Int J Environ Res Public Health.

[5] Phiri AFND, Abia ALK, Amoako DG, Mkakosya R, Sundsfjord A, Essack SY (2021). Burden, antibiotic resistance and clonality of *shigella spp*. Implicated in community-acquired acute diarrhea in Lilongwe, Malawi. Trop Med Infect Dis.

[6] Wenham M (2013). Dying for a drink in Turkana. Kenya, Practical action.

[7] Busienei PJ, Ogendi GM, Mokua MA (2019). Open defecation practices in Lodwar, Kenya a mixed-methods research. Environ Health Insights.

[8] Serwecinska L (2020). Antimicrobials and antibiotic-resistant bacteria: a risk to the environmnt and to public health. J water.

[9] Sosa AJ, Byarugaba DK, Ama CF, Hsueh Po-R, Kariuki S, Iruka NO (2010). Antimicrobial resistance in developing countries: poverty and root causes of resistance in developing countries.

[10] Shlla Bushra (2020). The gram stain. University of Mosul.

[11] Imen BS, Ridha M, Mahjoub A (2012). Laboratory typing methods for diagnostic of *Salmonella* strains the old organism that continued challenges. Research gate.

[12] National Health Service (NHS) (2018). UK standards for microbiological investigations: identification of *Salmonella* species.

[13] Centre of Disease Centre (2006). 10^th^ Annual pulsenet update meeting.

[14] Claudia A, Vander P, Vinas MR, Terragno R, Bruno SB, Binsztein N (2010). Laboratory protocol ''serotyping of shigella spp". WHO Global Foodborne Infections Network Laboratory Sub-Committee 2010.

[15] Mamuye Y, Metaferia G, Birhanu A, Desta K, Fantaw S (2015). Isolation and antibiotic susceptibility patterns of shigella and *Salmonella* among under five children with acute diarrhea. A cross-sectional study at selected public health facilities in Addis Ababa Ethiopia. J Clin Microbiology.

[16] Hayamo M, Alemayehu T, Tadesse B, Mitiku E, Bedawi Z (2021). Magnitude, risk factors and antimicrobial susceptibility pattern of *Shigella* and *Salmonella* among children with diarrhea in Southern Ethiopia: a cross-sectional study 2021. SAGE Open Med.

[17] Admassu M, Yemane G, Kibret M, Abera B, Nibret E, Adal M (2015). Prevalence and antibiogram of shigella and, *Salmonella spp* from under five children with acute diarrhea in Bahir Dar Town. Ethiop J Sci Technol.

[18] Mahyar A, Ayazi P, Kheirabi K, Sadeghi S, Daneshi-Khohan MM, Javadi A (2011). Antimicrobial resistance pattern of shigella species isolated in children with shigellosis. Pediatric Research.

[19] Thompson CN, Duy PT, Baker S (2015). The rising dominance of shigella sonnei: an intercontinental shift in etiology of bacillary dysentery 2015. PLoS Negl Trop Dis.

[20] Konate A, Guessennd N, Kouadio F, Dembele R, Kagambega A, Kouadio I (2019). Epidemiology and resistance phenotypes of *Salmonella spp*. Strains responsible for gastroenteritis in children less than five years of age in Ouagadougou, Burkina Faso. Arch Clin Microbiol.

